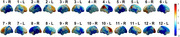# Interrogating the cognitive‐behavioural impairment in a motor‐dominant neurodegeneration (ALS), based on brain state dynamics of resting‐state EEG

**DOI:** 10.1002/alz.087312

**Published:** 2025-01-03

**Authors:** Marjorie Metzger

**Affiliations:** ^1^ Trinity Biomedical Sciences Institute, Trinity College Dublin, University of Dublin, Dublin, Dublin 2 Ireland

## Abstract

**Background:**

Amyotrophic lateral sclerosis (ALS) shares pathological and genetic underpinnings with frontotemporal dementia (FTD). ALS manifests with diverse symptoms, including progressive neuro‐motor degeneration, muscle weakness, but also cognitive‐behavioural changes in up to half of the cases. Resting‐state EEG measures, particularly spectral power and functional connectivity, have been instrumental for discerning abnormal motor and cognitive network function in ALS [1]–[3]. Based on our recent findings using EEG microstates [4], we hypothesized that dynamic analysis of time‐varying spectral EEG measures at source‐level can further interrogate the altered functional network that underpin the cognition and behaviour in ALS as a motor‐dominant neurodegeneration.

**Method:**

For this purpose, we aimed to identify transient brain states associated with specific functional networks and to characterise the spatio‐temporal and spectral alterations in these brain states using high‐density resting‐state EEG, recorded from 99 individuals with ALS and 78 healthy controls (HC). Using a time‐delay embedded Hidden‐Markov‐Model on source‐reconstructed EEG data [5], we identified transient and recurrent brain states characterised by spectral power and coherence. The model was trained to convert source‐reconstructed time courses into sequences of functional networks (brain states).

**Result:**

Twelve brain states were identified with distinct patterns of spectral power and coherence for individuals with ALS and HC. The Brain States 1, 3, 7 and 9 showed significant association between brain states fractional occupancy and behavioural decline (Beaumont Behavioural Inventory [7]; >0.25, q<0.03, >0.65), while state 5 showed association with fluency decline (Edinburgh Cognitive and Behavioural ALS scale [8]; = ‐0.3, q = 0.004, = 0.83). States 1, 7 and 10 were characterised by frontal lobe activation (spectral power higher than the average within the state), while state 3 exhibited activation in the sensorimotor network. Highest spectral power during state 5 was in the supplementary motor area, a region primarily associated with motor planning but also possibly associated with language [9].

**Conclusion:**

This study demonstrated the utility of resting‐state EEG and its temporal decomposition based on spatio‐spectral dynamics for investigating and interrogating the less‐dominant cognitive‐behavioural decline in ALS. It shows clear potentials for applications in cognate conditions such as FTD and Alzheimer’s disease with a dominant cognitive‐behavioural decline.